# Supplementary material for: Virtual lesions in MEG reveal increasing vulnerability of the language network from early childhood through adolescence
Source: Nat Commun. 2023 Nov 11;14:7313. doi: 10.1038/s41467-023-43165-7 (PMC10640569; doi:10.1038/s41467-023-43165-7)

Supplementary Table and Figures for:

Virtual lesions in MEG reveal increasing vulnerability of the language network from early childhood through adolescence

Brady J. Williamson[1], Hansel M. Greiner[2,3], Darren S. Kadis[4,5]

[1] Department of Radiology, University of Cincinnati, OH, United States
[2] Division of Neurology, Cincinnati Children's Hospital Medical Center, OH, United States
[3] Department of Pediatrics, College of Medicine, University of Cincinnati, OH, United States
[4] Neurosciences and Mental Health, Hospital for Sick Children, Toronto, ON, Canada
[5] Department of Physiology, University of Toronto, ON, Canada

Supplementary Table 1. Summary of Results from Attack Analyses.

Whole Brain Random Attacks

	Estimated df	*F*	*p*	*Functional R^2*	*sr*	95% CI contains 0
**Overall Model**	28.13	5.99	< 0.0001	0.013		
**Adjusted R^2 = 0.947**						
Intercept	18.91	2106.29	< 0.0001	NA	NA	NA
Age	**3.04**	**31.44**	**< 0.0001**	**0.004**	**0.03**	**No**
Sex	0.73	0.203	0.7540	0.0002	0.006	Yes
Handedness	2.46	3.05	0.0206	-0.0001	0.0007	Yes

Whole Brain Betweenness Centrality-Based Attacks

	Estimated df	*F*	*p*	*Functional R^2*	*sr*	95% CI contains 0
**Overall Model**	27.24	6.82	< 0.0001	0.082		
**Adjusted R^2 = 0.934**						
Intercept	117.69	984.42	< 0.0001	NA	NA	NA
Age	**4.25**	**126.38**	**< 0.0001**	**0.038**	**0.066**	**No**
Sex	1.273	1.583	0.345	0.016	0.013	Yes
Handedness	1.01	0.019	0.9070	0.0003	0	Yes

Stories Network Random Attacks

	Estimated df	*F*	*p*	*Functional R^2*	*sr*	95% CI contains 0
**Overall Model**	32.09	13.39	< 0.0001	0.017		
**Adjusted R^2 = 0.965**						
Intercept	18.55	77.17	< 0.0001	NA	NA	NA
Age	**4.59**	**44.51**	**< 0.0001**	**0.008**	**0.031**	**No**
Sex	0.0042	0.223	0.965	-0.0001	0.007	Yes
Handedness	1.00	0.62	0.4319	0.0009	0.019	Yes

Stories Network Betweenness Centrality-Based Attacks

	Estimated df	*F*	*p*	*Functional R^2*	*sr*	95% CI contains 0
**Overall Model**	30.08	6.75	< 0.0001	0.057		
**Adjusted R^2 = 0.915**						
Intercept	16.89	646.17	< 0.0001	NA	NA	NA
Age	**3.97**	**86.25**	**< 0.0001**	**0.028**	**0.0596**	**No**
Sex	3.390	5.084	0.0003	0.013	0.023	Yes
Handedness	2.83	12.72	< 0.0001	0.0008	0.019	Yes

* **Bold** indicates significance for independent variable in the multivariate modeling; *sr* = semipartial correlation.

Supplementary Figure 1. Mean Percolation Point by Density +/- standard deviation (shaded) for each attack strategy using the whole-brain parcellation for First and Fourth Age Quartiles (panels a,b,c) and for all participants (panels d,e,f). Source data are provided as a Source Data file.

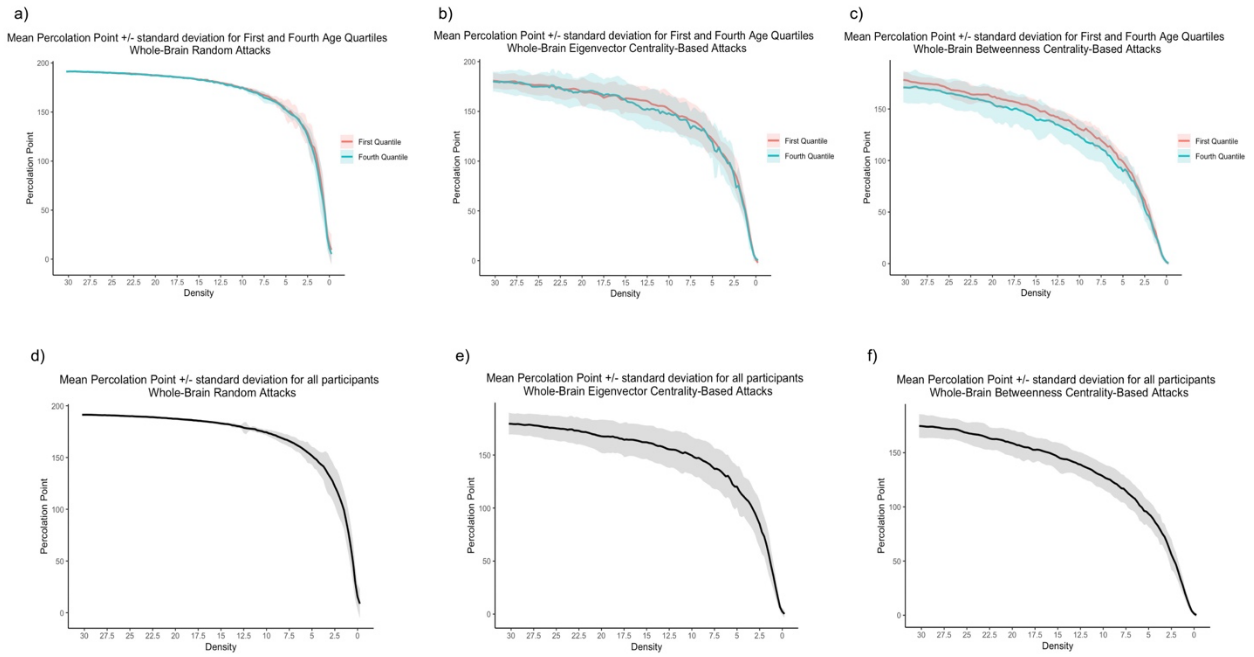

Supplementary Figure 2. Mean Percolation Point by Density +/- standard deviation (shaded) for each attack strategy using the stories network parcellation for First and Fourth Age Quartiles (panels a,b,c) and for all participants (panels d,e,f). Source data are provided as a Source Data file.

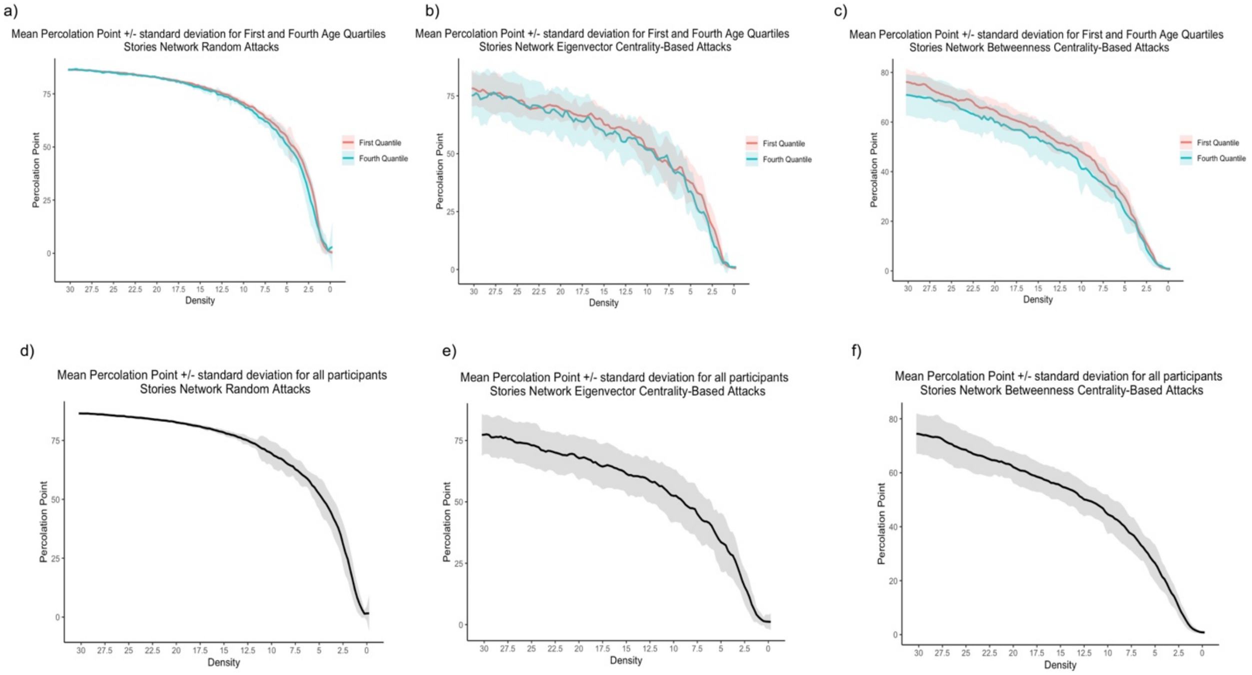

Supplementary Figure 3. Results for random attacks on the whole-brain parcellation. Data are presented as the beta estimate across densities +/- 95% CIs (shaded). The overall model was significant (see, Supplementary Table 1); beyond the Intercept (panel a), Age (negative, panel b) was the only significant regressor of interest ($F = 31.44$, $p < 0.0001$). Sex (panel c) and Handedness (panel d) were not significant due to the model p-value not meeting the threshold for significance (0.01) and/or 95% CIs contained 0 throughout the whole density range, indicating no results are significant. Source data are provided as a Source Data file.

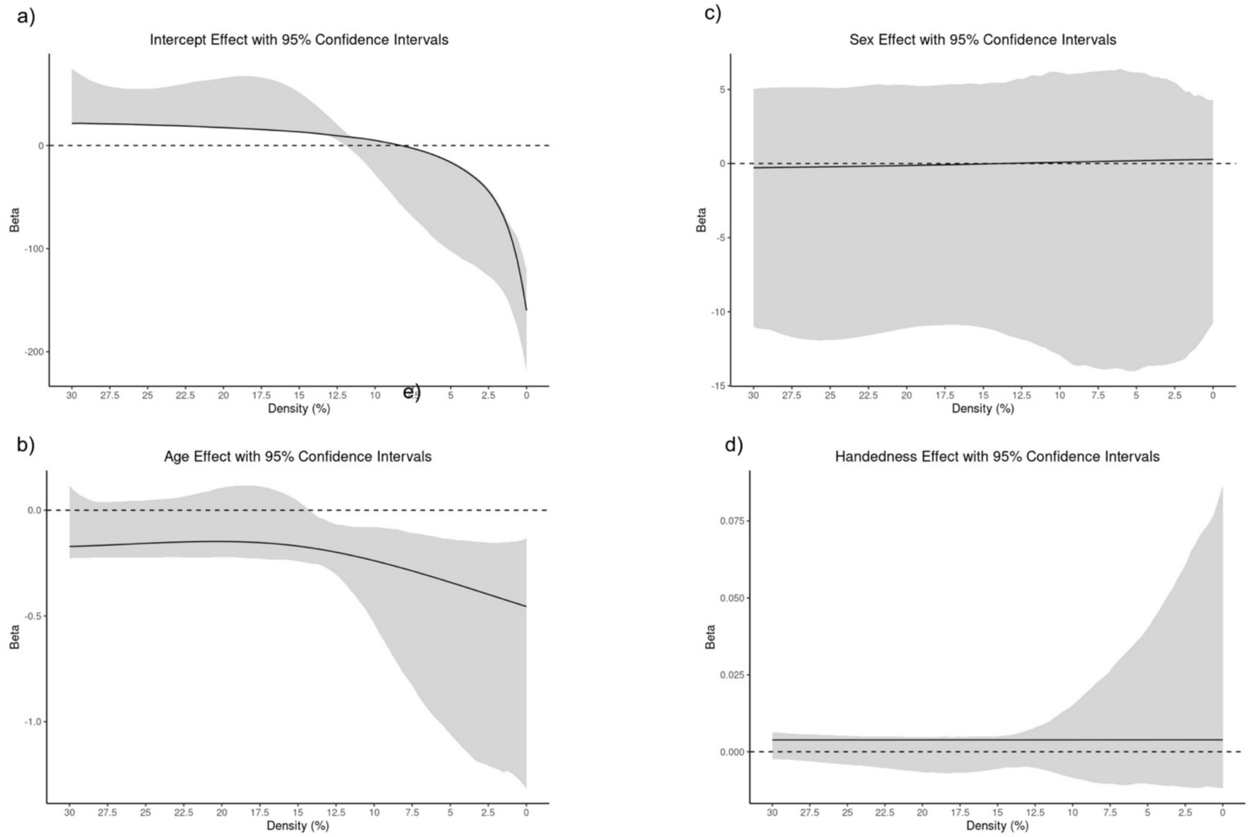

Supplementary Figure 4. Results for betweenness centrality-based attacks on the whole-brain parcellation. Data are presented as the beta estimate across densities +/- 95% CIs (shaded). The overall model was significant. Beyond the Intercept (panel a), Age (negative, panel b) was the only significant regressor of interest at all densities ($F = 126.38$, $p < 0.0001$). Sex (panel c) and Handedness (panel d) were not significant due to the model p-value not meeting the threshold for significance (0.01) and/or 95% CIs contained 0 throughout the whole density range. Source data are provided as a Source Data file.

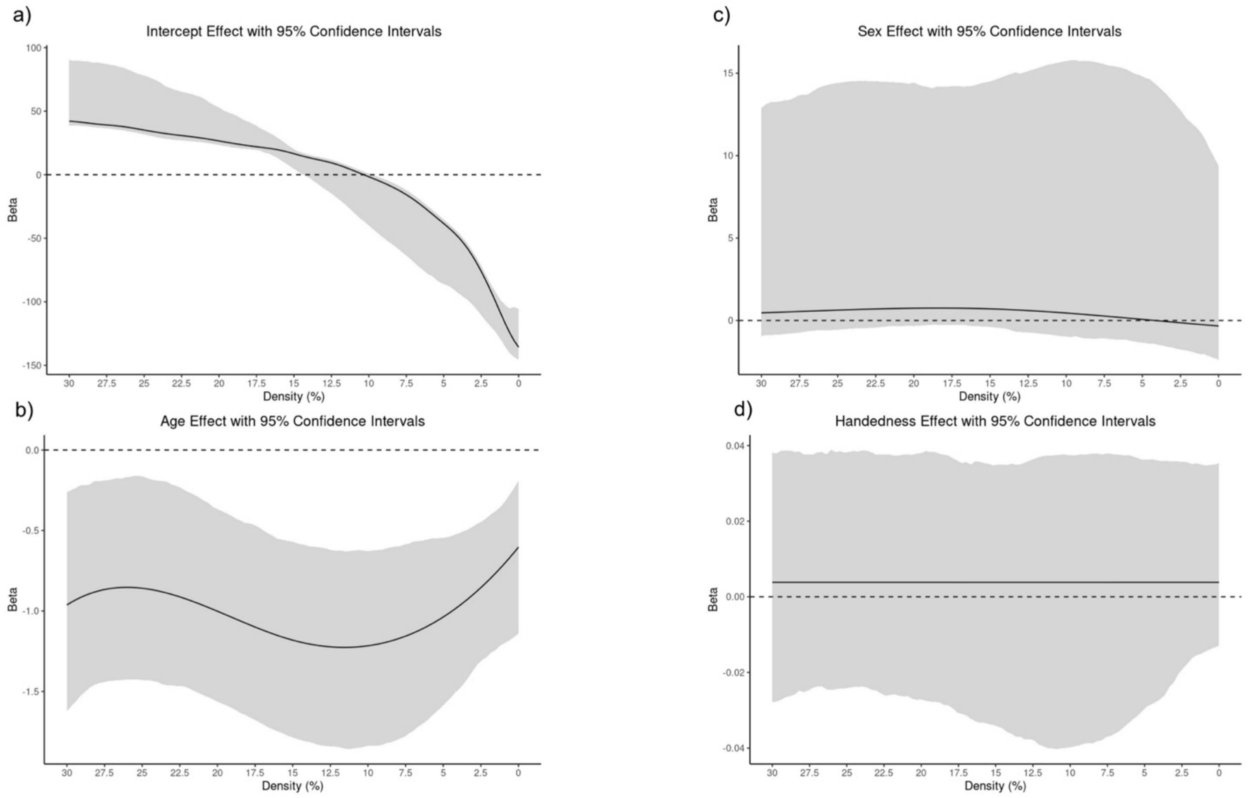

Supplementary Figure 5. Results for eigenvector centrality-based attacks on the whole-brain parcellation. Data are presented as the beta estimate across densities +/- 95% CIs (shaded). The overall model was not significant; only the Intercept was uniquely predictive (panel a). Age (panel b), Sex (panel c) and Handedness (panel d) were not significant due to the model p-value not meeting the threshold for significance (0.01) and/or 95% CIs contained 0 throughout the whole density range. Source data are provided as a Source Data file.

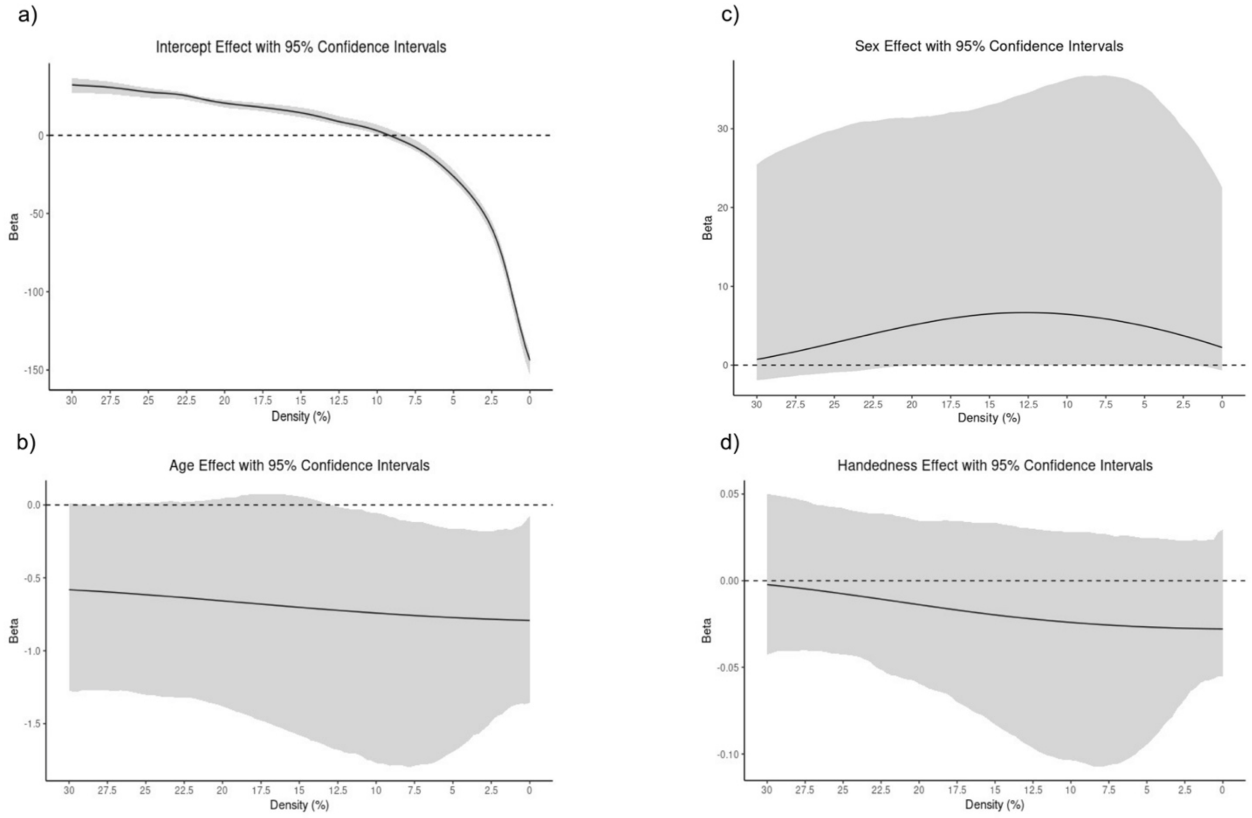

Supplementary Figure 6. Results for random attacks on the stories network parcellation. Data are presented as the beta estimate across densities +/- 95% CIs (shaded). The overall model was significant (see, Supplementary Table 1); beyond the Intercept (panel a), Age (negative, panel b) was the only significant regressor of interest ($F = 44.51$, $p < 0.0001$). Sex (panel c) and Handedness (panel d) were not significant due to the model p-value not meeting the threshold for significance (0.01) and/or 95% CIs contained 0 throughout the whole density range. Source data are provided as a Source Data file.

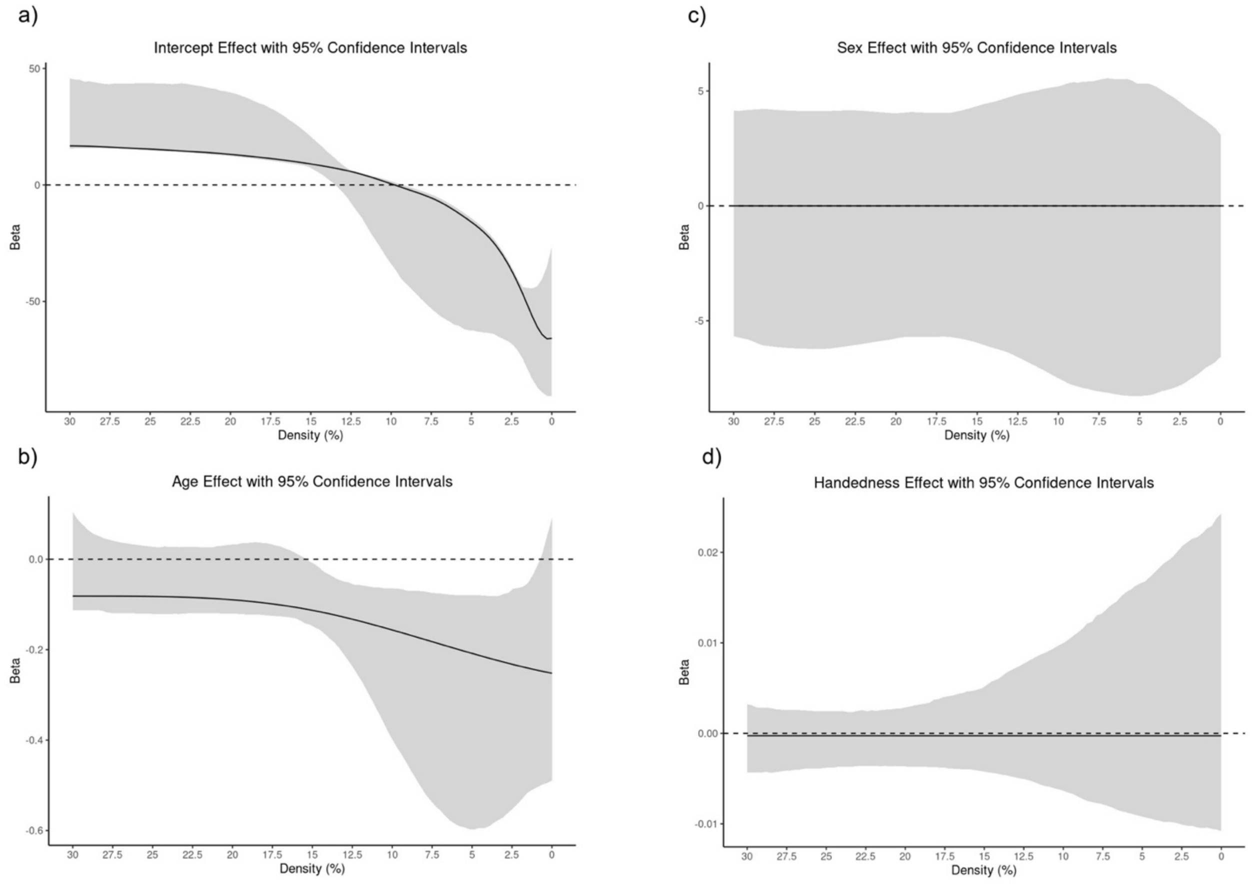

Supplementary Figure 7. Results for betweenness centrality-based attacks on the stories network parcellation. Data are presented as the beta estimate across densities +/- 95% CIs (shaded). The overall model was significant (see, Supplementary Table 1); beyond the Intercept (panel a), Age (negative, panel b) was the only significant regressor of interest at all densities ($F = 86.25$, $p < 0.0001$). Sex (panel c) and Handedness (panel d) were not significant due to the model p-value not meeting the threshold for significance (0.01) and/or 95% CIs contained 0 throughout the whole density range. Source data are provided as a Source Data file.

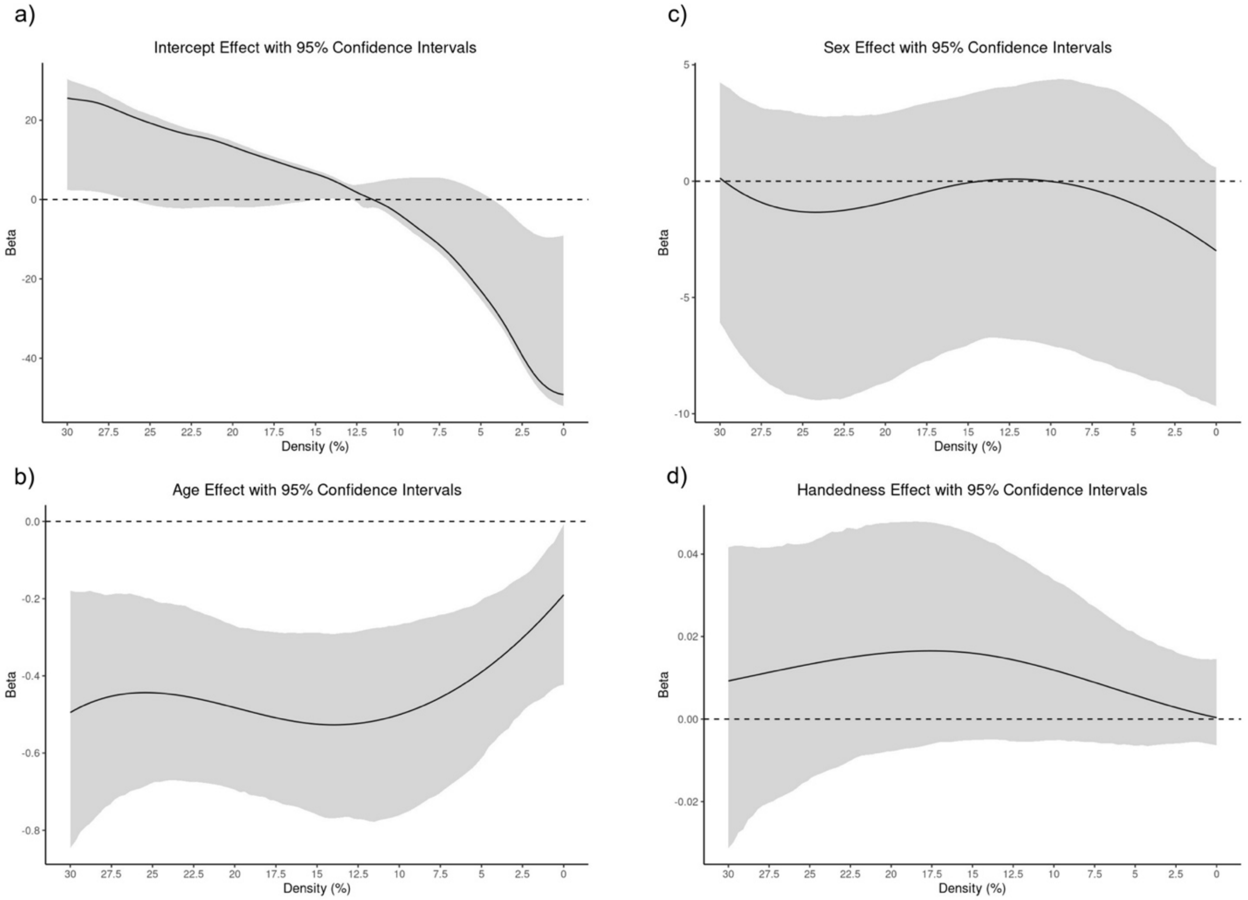

Supplementary Figure 8. Results for eigenvector centrality-based attacks on the stories network parcellation. Data are presented as the beta estimate across densities +/- 95% CIs (shaded). The overall model was not significant; only the Intercept was uniquely predictive (panel a). Age (panel b), Sex (panel c) and Handedness (panel d) were not significant due to the model p-value not meeting the threshold for significance (0.01) and/or 95% CIs contained 0 throughout the whole density range, indicating no results are significant. Source data are provided as a Source Data file.

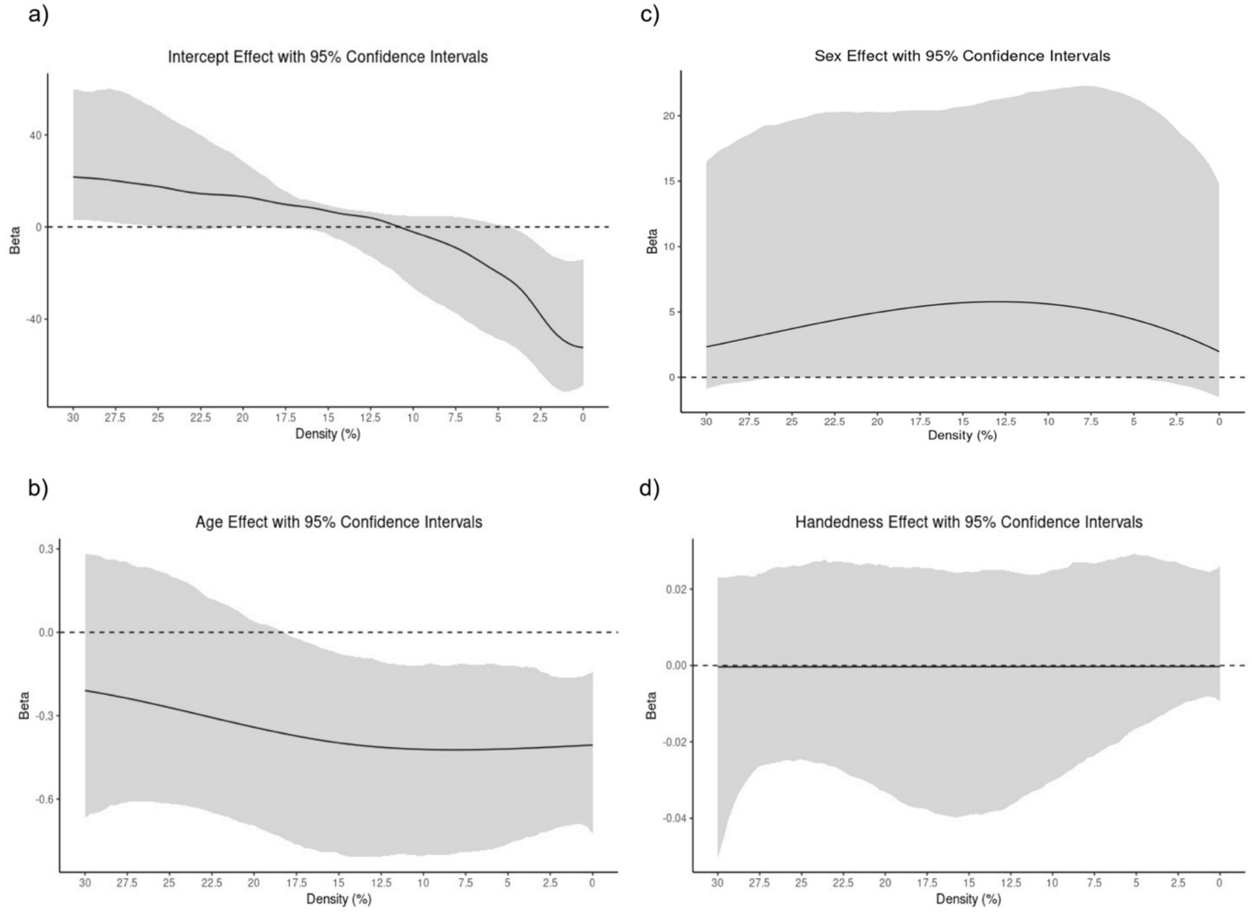

Supplementary Figure 9. Distribution of nodes removed in the whole-brain (panels a and b) and stories network (panels c and d) for betweenness centrality-based attacks for the 1st (panels a and c) and 4th (panels b and d) quartiles of age. Regions are colored by the percentage of participants for which the node was removed prior to network failure (dark to light). These results were displayed for 2.5% density across participants. Brighter colors indicate that the node was removed in more participants, implying group-level criticality to the network.

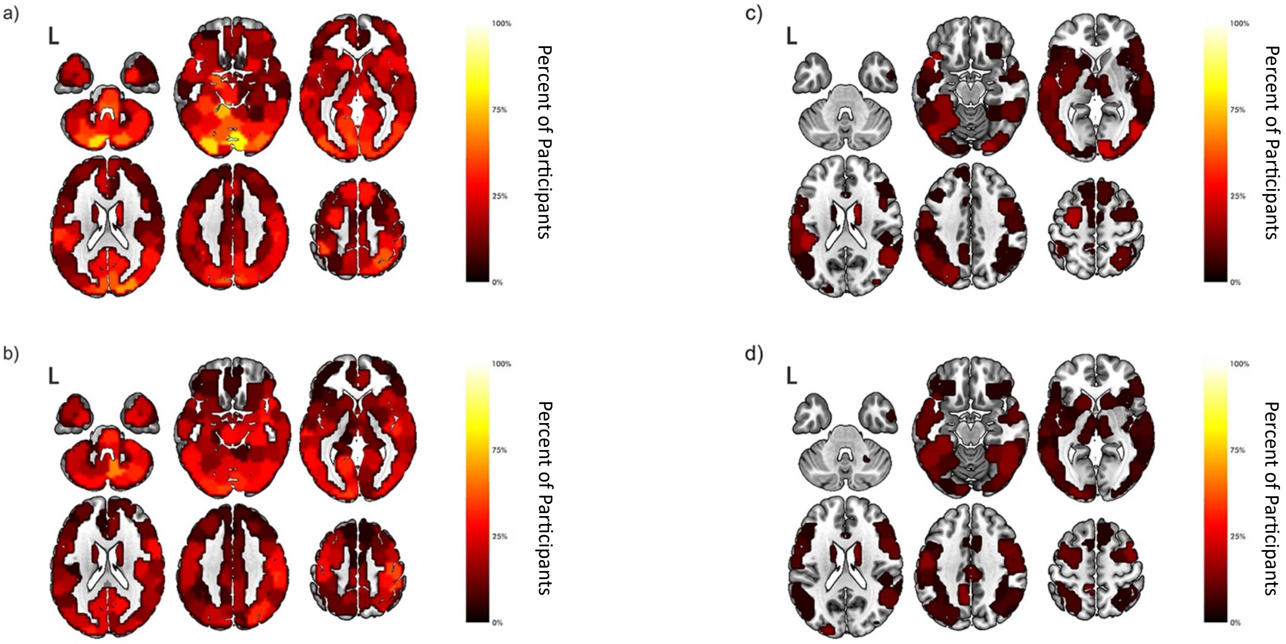

Supplementary Figure 10. Distribution of nodes removed in the whole-brain (panel a and b) and stories network (panels c and d) for betweenness centrality-based attacks for the 1st (panels a and c) and 4th (panels b and d) quartiles of age. Regions are colored by the percentage of participants for which the node was removed prior to network failure (dark to light). These results were displayed for 7.5% density across participants. Brighter colors indicate that the node was removed in more participants, implying group-level criticality to the network.

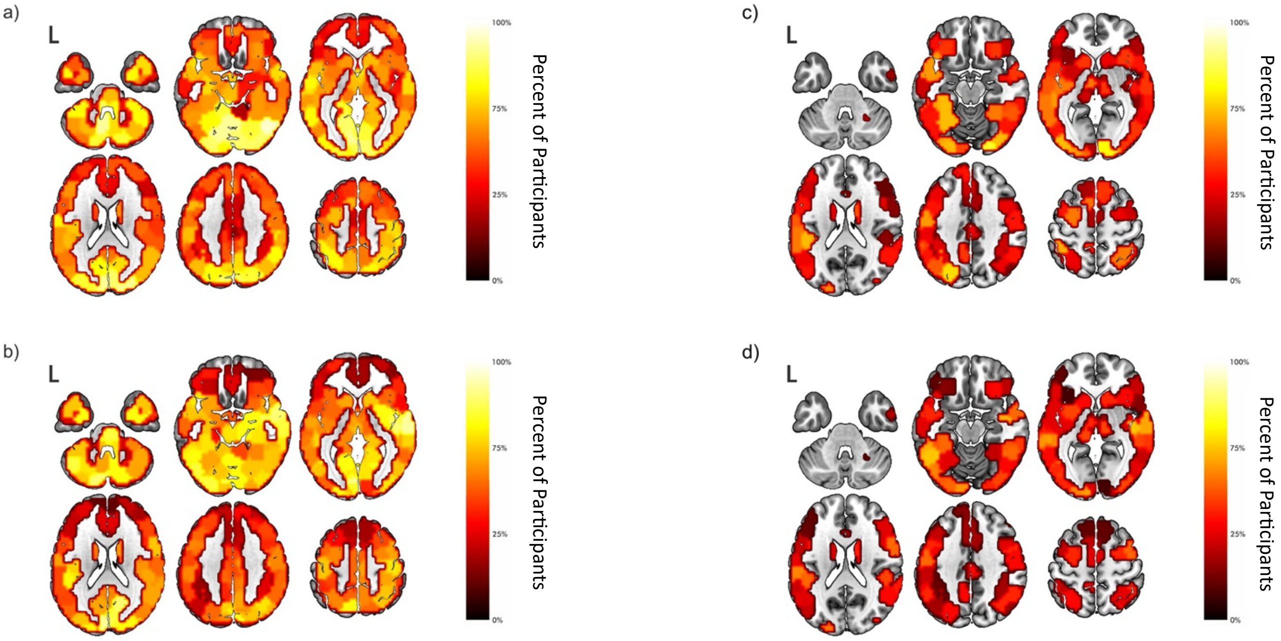

Supplement: Supplementary file 1 — Supplementary Information [file 41467_2023_43165_MOESM1_ESM.pdf]
