# Supplementary material for: Virtual lesions in MEG reveal increasing vulnerability of the language network from early childhood through adolescence
Source: Nat Commun. 2023 Nov 11;14:7313. doi: 10.1038/s41467-023-43165-7 (PMC10640569; doi:10.1038/s41467-023-43165-7)
Supplement: Supplementary file 3 — Reporting Summary [file 41467_2023_43165_MOESM3_ESM.pdf]

nature portfolio

Corresponding author(s): Darren S. Kadis

Last updated by author(s): Sep 27, 2023

Reporting Summary

Nature Portfolio wishes to improve the reproducibility of the work that we publish. This form provides structure for consistency and transparency in reporting. For further information on Nature Portfolio policies, see our Editorial Policies and the Editorial Policy Checklist.

Statistics

For all statistical analyses, confirm that the following items are present in the figure legend, table legend, main text, or Methods section.

n/a	Confirmed	
☐	☒	The exact sample size (*n*) for each experimental group/condition, given as a discrete number and unit of measurement
☐	☒	A statement on whether measurements were taken from distinct samples or whether the same sample was measured repeatedly
☐	☒	The statistical test(s) used AND whether they are one- or two-sided<br>*Only common tests should be described solely by name; describe more complex techniques in the Methods section.*
☐	☒	A description of all covariates tested
☐	☒	A description of any assumptions or corrections, such as tests of normality and adjustment for multiple comparisons
☐	☒	A full description of the statistical parameters including central tendency (e.g. means) or other basic estimates (e.g. regression coefficient) AND variation (e.g. standard deviation) or associated estimates of uncertainty (e.g. confidence intervals)
☐	☒	For null hypothesis testing, the test statistic (e.g. *F*, *t*, *r*) with confidence intervals, effect sizes, degrees of freedom and *P* value noted<br>*Give P values as exact values whenever suitable.*
☒	☐	For Bayesian analysis, information on the choice of priors and Markov chain Monte Carlo settings
☐	☒	For hierarchical and complex designs, identification of the appropriate level for tests and full reporting of outcomes
☒	☐	Estimates of effect sizes (e.g. Cohen's *d*, Pearson's *r*), indicating how they were calculated

Our web collection on statistics for biologists contains articles on many of the points above.

Software and code

Policy information about availability of computer code

Data collection	MRI data were collected using standard software provided by the vendor, Philips MR release 5.1/5.6. MEG data were collected using standard software provided by the vendor, CTF Acq 5.4.2.
Data analysis	Routine MRI and MEG preprocessing and connectivity analyses were carried out in MATLAB (R2022b) using SPM12 (https://www.fil.ion.ucl.ac.uk/spm/software/spm12/) and Fieldtrip (Oostenveld, Fries, Maris, & Schoffelen, 2011, Computational Intelligence and Neuroscience) routines. Graph analyses were performed using Brain Connectivity Toolbox functions (https://sites.google.com/site/bctnet/; Rubinov & Sporns, 2010, NeuroImage), also in MATLAB. Functional data analysis (FDA) was performed in R 4.1.0 using the fda and refund packages, version 5.1.9 and 0.1-30 respectively. Our code has been deposited in a github repository with relevant instructions for reproducing findings: https://github.com/willi3by/MEG_FDA .

For manuscripts utilizing custom algorithms or software that are central to the research but not yet described in published literature, software must be made available to editors and reviewers. We strongly encourage code deposition in a community repository (e.g. GitHub). See the Nature Portfolio guidelines for submitting code & software for further information.

Data

Policy information about availability of data

All manuscripts must include a data availability statement. This statement should provide the following information, where applicable:

- Accession codes, unique identifiers, or web links for publicly available datasets
- A description of any restrictions on data availability
- For clinical datasets or third party data, please ensure that the statement adheres to our policy

The percolation point by density data (.mat files) generated in this study, along with minimal linked demographic data, have been deposited in the github repository (https://github.com/willi3by/MEG_FDA). The raw MEG and MRI data are protected under privacy laws. The data hosted on github are also provided in the Source Data file.

Research involving human participants, their data, or biological material

Policy information about studies with human participants or human data. See also policy information about sex, gender (identity/presentation), and sexual orientation and race, ethnicity and racism.

Reporting on sex and gender	Sex (parent-report for children under 18.0 years, self-report for those 18.0 years and older) was reported, and considered in our analyses (i.e., as a covariate of interest).
Reporting on race, ethnicity, or other socially relevant groupings	Race and ethnicity were not considered in the current analyses.
Population characteristics	The primary focus of this study was to characterize age-related changes in language network resilience, in childhood. As such, children age 5 years to less than 19 years, were studied.
Recruitment	Children were recruited from the community (Cincinnati area), through fliers posted at Cincinnati Children's Hospital Medical Center, and through a list of potential research participant families, maintained by the Hospital.
Ethics oversight	IRB was obtained at Cincinnati Children's Hospital Medical Center (primary research site); REB was obtained at the Hospital for Sick Children (analysis site).

Note that full information on the approval of the study protocol must also be provided in the manuscript.

Field-specific reporting

Please select the one below that is the best fit for your research. If you are not sure, read the appropriate sections before making your selection.

☒ Life sciences ☐ Behavioural & social sciences ☐ Ecological, evolutionary & environmental sciences

For a reference copy of the document with all sections, see nature.com/documents/nr-reporting-summary-flat.pdf

Life sciences study design

All studies must disclose on these points even when the disclosure is negative.

Sample size	The current analyses reflect secondary use of data collected under two other projects, funded by the Research Institute at Cincinnati Children's Hospital Medical Center and the National Institute of Neurological Disorders and Stroke (NINDS) at the National Institutes of Health (NIH; award R21NS106631). The former was pilot study, establishing the potential for connectivity-based mapping to identify critical language sites in childhood. The NIH-funded study was powered to detect developmental changes in language lateralization using connectivity- and network-based mapping; based on previous literature, we anticipated power of 0.73 to 0.98, at n=50 and alpha=0.05.
Data exclusions	Of the 85 datasets available to us, only 4 were excluded, due to poor data quality (total movement greater than 5mm for the MEG stories-listening acquisition).
Replication	To maximize reproducibility, we used open-source tools with detailed documentation of parameters to promote transparency. Any parameters that needed to be set for analyses were determined in a data-driven process to reduce bias. Statistical thresholds were adequately conservative, corrected where needed (i.e., we used permutation testing). All procedures used to generate these results could be exactly repeated given the correct equipment (i.e., MEG and MRI).
Randomization	This is not relevant to the current study as there were no experimental groups. This study focused on MEG-based brain function in a cohort of typically-developing children.
Blinding	Blinding was not relevant to this study as there was no intervention / treatment.

Reporting for specific materials, systems and methods

We require information from authors about some types of materials, experimental systems and methods used in many studies. Here, indicate whether each material, system or method listed is relevant to your study. If you are not sure if a list item applies to your research, read the appropriate section before selecting a response.

Materials & experimental systems

n/a	Involved in the study
x	Antibodies
x	Eukaryotic cell lines
x	Palaeontology and archaeology
x	Animals and other organisms
x	Clinical data
x	Dual use research of concern
x	Plants

Methods

n/a	Involved in the study
x	ChIP-seq
x	Flow cytometry
	x MRI-based neuroimaging

Magnetic resonance imaging

Experimental design

Design type: Only structural images were obtained; used for headmodeling / source localization of MEG data

Design specifications: N/A

Behavioral performance measures: N/A

Acquisition

Imaging type(s): Structural (3D T1-weighted MRI)

Field strength: 3T

Sequence & imaging parameters: 3D T1-weighted images (1.0×1.0×1.0mm voxels), MDEFT sequence

Area of acquisition: whole head ("air-to-air")

Diffusion MRI: [ ] Used [x] Not used

Preprocessing

Preprocessing software: SPM12 (https://www.fil.ion.ucl.ac.uk/spm/software/spm12/) routines were used for MRI segmentation and warping of template (MNI space) source positions into individual space. MEG preprocessing (MRI-MEG registration, filtering, epoching) was carried out in FieldTrip (Oostenvelt et al., 2011, Computational Intelligence and Neuroscience; August 21, 2020 release). Both SPM12 and FieldTrip ran in MATLAB R2022b (MathWorks).

Normalization: Template source positions (ICBM152) were nonlinearly warped to individual space using SPM12 normalization ('new' routine).

Normalization template: ICBM152

Noise and artifact removal: N/A (only structural MR images used in this study)

Volume censoring: N/A

Statistical modeling & inference

Model type and settings: No statistical modeling was performed with data derived from MRI

Effect(s) tested: N/A, see above

Specify type of analysis: [x] Whole brain [ ] ROI-based [ ] Both

Statistic type for inference (See Eklund et al. 2016): N/A, see above

Correction: N/A, see above

Models & analysis

n/a	Involved in the study
☒	☐ Functional and/or effective connectivity
☒	☐ Graph analysis
☒	☐ Multivariate modeling or predictive analysis
